# Severe adverse reactions to benzathine penicillin G in rheumatic heart disease: A systematic review and meta-analysis

**DOI:** 10.1371/journal.pone.0322873

**Published:** 2025-05-07

**Authors:** Eshetie Melese Birru, Kefyalew Addis Alene, Laurens Manning, Kevin T. Batty, Brioni R. Moore

**Affiliations:** 1 Curtin Medical School, Curtin University, Bentley, Western Australia; 2 Department of Pharmacology, School of Pharmacy, College of Medicine and Health Sciences, University of Gondar, Gondar, Ethiopia; 3 School of Population Health, Faculty of Health Sciences, Curtin University, Bentley, Western Australia; 4 Geospatial and Tuberculosis Research Team, The Kids Research Institute Australia, Nedlands, Western Australia; 5 Medical School, The University of Western Australia, Crawley, Western Australia; 6 Wesfarmers Centre of Vaccines and Infectious Diseases, The Kids Research Institute Australia, Nedlands, Western Australia; 7 Curtin Medical Research Institute, Curtin University, Bentley, Western Australia; Concordia University Irvine, UNITED STATES OF AMERICA

## Abstract

**Background:**

Fear of severe adverse reaction (SAR) and reluctance of health care providers to administer intramuscular injections are major contributing factors to poor adherence of benzathine penicillin G (BPG) in the management of rheumatic heart disease (RHD). However, data on the risk of SARs following BPG injections for RHD are relatively limited and inconclusive. Our systematic review and meta-analysis aimed to evaluate the incidence of SARs associated with BPG injections used for secondary prophylaxis of RHD.

**Methods:**

A systematic literature search of PubMed, Scopus and Web of Science databases was conducted to identify relevant studies reporting adverse reactions following BPG injections in patients with acute rheumatic fever (ARF) and/or RHD. A random effect meta-analysis was performed to estimate the pooled incidence of SARs.

**Result:**

Nine studies (eight cohort and one randomized controlled trial), comprising 11,587 participants and > 154,760 BPG injections, were included in the analysis. The pooled incidence of SARs was 9.7 per 10,000 cases (95% CI: 0.1–29.2) and 1.1 per 10,000 BPG injections (95% CI: 0.4–2.2). Six fatal reactions were reported (0.05% of patients and 24% of SARs), all occurring in patients with severe RHD.

**Conclusion:**

SARs following BPG injections in patients with ARF or RHD are rare. Our findings highlight the importance of balancing the low rate of SARs against the benefits of BPG in secondary prophylaxis for RHD, particularly in high-risk populations. High-quality longitudinal research and comprehensive adverse reaction reporting are essential to address safety concerns among healthcare providers and patients that impact BPG delivery.

## Introduction

Acute rheumatic fever (ARF), a preventable autoimmune condition triggered by group A Streptococcal infection, remains a significant contributor to global cardiovascular morbidity through its progression to rheumatic heart disease (RHD) [1]. Worldwide, over 40 million people are affected by ARF/RHD, with the greatest burden occurring in socioeconomically disadvantaged countries [2]. Even in high income settings, where healthcare systems are advanced, the burden of RHD remains high among underprivileged populations [3,4].

Benzathine penicillin G (BPG) is a long-acting formulation designed for sustained release of the active penicillin moiety after intramuscular injection [5]. Since the 1950s, BPG has remained the recommended first-line medication for primary and secondary prophylaxis of ARF and RHD, due to its effectiveness, availability, and affordability [6–8].

To prevent disease recurrence, reduce disease progression and improve patient outcomes, high adherence (at least 80% of the scheduled annual injections) to BPG injections given every two to four weeks is desirable [9,10]. However, it is widely acknowledged that BPG adherence for secondary prophylaxis is suboptimal [3,11], impacting the management of RHD patients, particularly in resource-limited settings [8,12]. Several factors contribute to suboptimal BPG therapy [6,13], with safety concerns being one of the most commonly reported issues [6,14–16].

The fear of sudden, severe, and potentially fatal reactions following BPG injection for treatment of RHD has been shown to result in healthcare providers feeling uncomfortable, and often reluctant, to administer BPG [14–16]. Although previous reports indicate that the incidence of confirmed anaphylaxis following BPG injection is low [17,18], anecdotal evidence from RHD endemic settings suggests higher rates [19]. Fatal reactions following BPG injections are particularly concerning because they are often memorable, and historical anecdotes can lead healthcare providers to oppose administering BPG [15,16]. In response to heightened concerns over severe adverse reactions (SARs), some endemic settings have either discontinued, restricted, or prohibited the use of BPG [20,21]. However, both recent studies and anecdotal evidence suggest that most BPG related SARs may be related to the severity of RHD or underlying cardiac issues, rather than to drug related anaphylaxis [14,22]. In light of this emerging evidence and its implications for BPG clinical use, experts recently recommend consideration of oral prophylaxis, preferably with penicillin, for high-risk RHD patients when it is reliably available and affordable [15].

There are few reports on the risk of adverse reactions to BPG in patients with RHD [14,23], and a paucity of systematic reviews and meta-analysis on this important issue. Similarly, systematic reviews [24,25] focusing on BPG safety when delivered for prevention of congenital syphilis have highlighted a scarcity of high-quality adverse event data. Our systematic review and meta-analysis sought to estimate the incidence of SARs, including fatal reactions, among ARF/RHD patients receiving BPG injections, based on eligible studies meeting the inclusion criteria.

## Methods

### Protocol registration

The current review was registered on International Prospective Register of Systematic Reviews (PROSPERO), registration number: CRD42024563774 and conducted in adherence to the Preferred Reporting Items for Systematic Reviews and Meta-Analyses (PRISMA) guidelines [26] (S1 Checklist*: PRISMA Checklist*).

### Eligibility criteria

Eligible studies included randomized controlled trials (RCTs) or cohort studies that assessed SARs following BPG injections in patients with ARF or RHD, where at least one SAR was documented, or explicit textual confirmation of their absence reported. However, the search strategy included all studies reporting adverse drug reactions following BPG injection, to ensure capture of all reported SARs. For the purpose of the search strategy, an adverse drug reaction was defined as “an appreciably harmful or unpleasant reaction resulting from an intervention related to the use of a medicinal product; adverse effects usually predict hazard from future administration and warrant prevention, or specific treatment, or alteration of the dosage regimen, or withdrawal of the product” [27] whilst SAR was defined as an adverse event that could be “life-threatening, cause disabilities, or result in prolonged hospital stays, hospitalization, or the need for intensive medical care” [28]. Both allergic and non-allergic SARs reported following BPG injection were included. All anaphylactic or anaphylactic-type reactions were classified as SARs. By contrast, other adverse events were only defined as SARs if documented as ‘severe’ or ‘fatal’ event(s) in the respective studies. Studies published in languages other than English were excluded. If multiple studies shared the same dataset or cohort, the study with the largest sample size was selected for inclusion.

### Information source and search strategy

Systematic literature searches were conducted in PubMed, Scopus, and Web of Science from the inception of these databases until 6 July 2024, to identify articles reporting SARs following BPG injection among ARF/RHD patients. The Medical Subject Heading (MeSH) terms and a combination of keywords related to BPG, ARF, and adverse reactions were used for the search (S1 File*: Search Strategy*). Forward and backward citation searches on the retrieved articles were conducted in Google and Google Scholar to identify additional relevant studies.

### Study selection and data extraction

Two independent reviewers (EMB and KAA) conducted title and abstract screening, and identified relevant studies based on predefined inclusion criteria using Rayyan, a web-based screening tool [29]. Any discrepancies were resolved through consensus and independent reviewers (BRM, KTB) were available for resolution, if required. A data extraction database (Microsoft Excel, version 365) was created to assemble predetermined relevant information from each publication; bibliographic details (name of first author, year of publication, year of data collection/review, and country), study characteristics (study design, sample size by cases and BPG injections, follow up duration), patient details (sex/male proportion, age or mean/median age, BPG exposure history, severity of RHD), and details of reported SAR (onset, intervention, outcomes). One author (EMB) extracted the data from full-text articles, whilst another (KAA) confirmed the extracted information.

### Risk of bias and quality of evidence assessment

To assess risk of bias and methodological quality in included studies, the Cochrane Risk of Bias Tool for the randomized clinical trial [30] and Newcastle-Ottawa quality scale [31] for cohort studies was used.

### Data synthesis and analysis

Narrative syntheses were conducted using all included studies to describe the outcomes of the studies. Random-effect meta-analyses were performed to estimate the pooled incidence of SARs (i.e., per 10,000 cases and 10,000 BPG injections) following BPG injection in RHD patients with 95% confidence interval. A pooled estimate of the incidence of SARs was calculated as a weighted average of adverse reactions estimated in the individual studies [32]. Heterogeneity among the included studies was evaluated using Higgins’ I² statistics [33], and potential sources of heterogeneity were investigated through subgroup and sensitivity analyses. Publication bias was evaluated visually using a funnel plot [34] and statistically using Egger’s test [35]. A sensitivity analysis was conducted to determine whether any specific study had a significant influence on the pooled incidence of SARs following BPG injection in patients with RHD/ARF. This analysis involved excluding low-quality studies and those with small sample sizes to assess their impact on the overall estimated incidence. Random effect meta-analyses were also conducted to estimate the incidence of fatal reactions following BPG injection. All data analysis was conducted using Stata Software version 17 (Stata Corp LLC, College Station, Texas 77845, USA).

## Results

### Study selection

**Fig 1:**
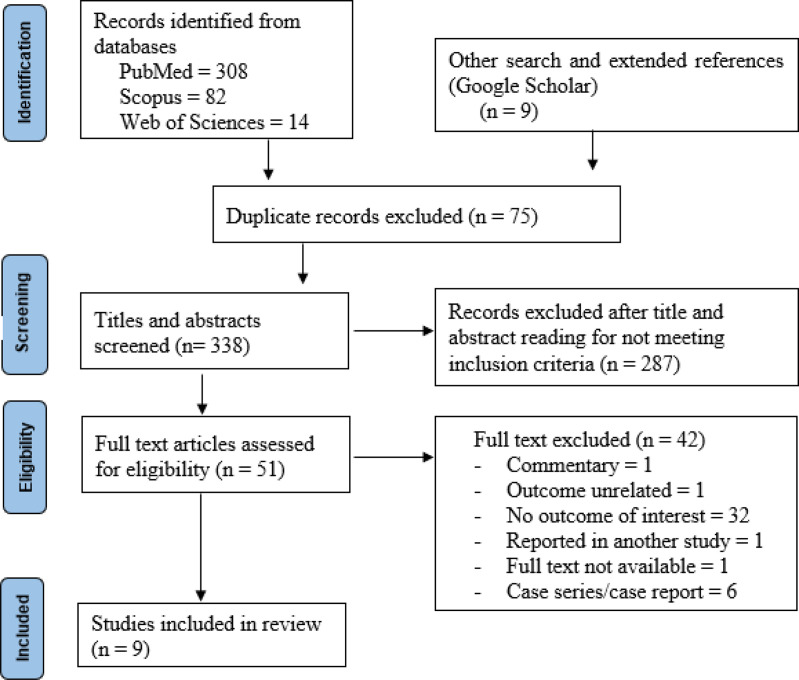
PRISMA flow diagram showing literature search and study selection.

Our systematic search identified 404 articles along with 9 additional articles from forward and backward searches in Google Scholar (Fig 1). After removing duplicates, 338 articles remained. Screening of titles and abstracts narrowed this to 51 for full text assessment, of which 9 met the inclusion criteria and were included in the review [20,22,23,36–41]. A list of the 338 studies remaining after duplicate removal, including those from extended reference searches, as well as the excluded articles with reasons for exclusion after full-text screening, is provided as a supplementary document (S1 Data *Excel: List of all literatures with reason for exclusion*).

### Study characteristics

The analysis encompassed nine studies [20,22,23,36–41], collectively involving a total of 11,587 participants. Sample sizes across these studies varied substantially, ranging from 32 to 4,712 participants (Table 1). The studies were conducted between 1955–2022 in at least 11 countries (one study was conducted across multiple countries), with over half of the studies being published after 2000. The study populations varied; three studies comprised adults and children, five focussed exclusively on children and one exclusively on adults. Among the six studies that reported participants’ sex, the proportion of male participants ranged from 41% to 66%. The follow-up period of the studies ranged from one month to six years. In all studies, patients received BPG doses of either 0.6 or 1.2 million international units (1MIU = 600 mg) administered every two to four weeks. A total of 154,760 BPG injections were reported across the six studies that provided information on the number of injections delivered [20,23,36,37,39,41]. Three studies did not clearly present or report the number of injections used in the respective study [22,38,40].

**Table 1:** Characteristics of included studies in the systematic review and meta-analyses.

Study first author	Year of publication	Country	Study design	Year of data collection	Follow up duration	Study population	Sample size	Number of injections	Male proportion (%)	Mean/median age (year)
Stollerman [36]	1955	USA	Prospective cohort	1952-1954	1-2 years	Children	410	4,871	NR	NR
Hsu [40]	1958	USA	Prospective cohort	1955-1957	1-27 months	Adults	32	NA	NR	20-54
Lue [39]	1975	Taiwan	Prospective cohort	1967-1971	6 months-6 years	Children	102	4,056	56	< 18
Markowitz [23]	1991	Multi-country^†^	Prospective cohort	1988-1990	6-24 months	Adults and children	1790	32,430	NR	5-28
Regmi [37]	2011	Nepal	Retrospective cohort	2007-2010	33 months	Adults and children	4712	77,300	46	NR
Mehta [38]	2016	India	Prospective cohort	2012-2015	6-18 months	Children	436	NR	66	12.2
Ali [22]	2018	Sudan	Mixed^‡^	2014–2018^†^	13-15 months^§^	Children	818	NR^*^	51	3-18
Bhat [41]	2021	India	Retrospective cohort	2015-2020	6 years	Adults and children	2878	25,819	41	31.54
Beaton [20]	2022	Uganda	RCT	2018 -2020	24 months	Children	409	10,284	43	12.6

NR: Not reported; RCT: Randomized controlled trial; ^†^ was conducted in 11 countries, ^‡^ has both retrospective and Prospective approaches; ^§^ the data collection period also looks 2012–2018, ^*^ Not reported due to unclear or unspecified data. The treatment regimen was either 0.6 MIU or 1.2 MIU BPG every 2–4 weeks depending on local guidelines.

### Quality and risk of bias assessment of studies included in the analysis

The quality assessment scores for the cohort studies ranged from three to six points, with a median score of five and an interquartile range (IQR) of three. Out of the eight cohort studies, six were rated as moderate quality (scores between five and six), while the remaining two were rated as low quality (score of three), according to the Newcastle-Ottawa quality rating scale (S2 File*: Quality assessment*). Moreover, the overall risk of bias assessment of the RCT study demonstrated a high risk of bias in one of the domains based on the Cochrane risk of bias assessment tool (S3 File*: Risk of bias assessment).*

### Severe adverse reactions

A total of 25 SARs were reported across the nine included studies (Table 2). Of the 25 SARs, 19 were classified as anaphylactic. The remaining cases included three non-allergic fatal reactions, two instance of severe serum sickness, and one case of sciatic nerve injury. Two studies, Ali et al. [22] and Mehta et al. [38] reported only fatal reactions following BPG injection, with no data on non-fatal severe or allergic adverse reactions reported.

**Table 2:** Allergic, SARs, anaphylactic and fatal reactions following BPG injection among ARF and RHD patients.

Study	Sample size^*^	BPG injections delivered	Allergic reactions	SARs	Anaphylaxis	Death	Onset and description of SAR, if reported	BPG product used
Stollerman (1955) [36]	410	4,871	5	1	0	0	Severe “serum sickness” type of reaction consisting of fever, angioneurotic oedema, and polyarthralgia	Bicillin
Hsu (1958) [40]	32	NR	6	2	1	1	Anaphylaxis within few minutes (n = 1, 20^th^ dose) and severe serum sickness (n = 1, first dose)	NR
Lue (1975) [39]	102	4,056	7	2	2	0**	NR	NR
Markowitz (1991) [23]	1,790	32, 430	57	4	4	1	All were anaphylaxis reactions (within minutes), All were on regular BPG	NR
Regmi (2011) [37]	4,712	77,300	65	5	5	0	NR	Lyophilized powder
Mehta (2016) [38]^†^	436	NR	1^§^	1^§^	1	1	The patient was on BPG	NR
Ali (2018) [22]^†^	818	NR	NR	3^§^	0	3	Within few minutes, one was new	Lyophilized powder
Bhat (2021) [41]	2,878	25,819	7	5	5	0	All within 2–10 minutes BPG exposure history (new, n = 4)	Lyophilized powder
Beaton (2022) [20]	409	10,284	8	2	1	0	Anaphylactic reaction (n = 1, within 3 minutes), Sciatic nerve injury (n = 1)	Lyophilized powder
Total	11,587	154,760^¶^	156	25	19	6		

*The sample size only refers patients who were taking BPG in the respective studies; † Only fatal reaction(s) reported, no information available about non-fatal allergic (severe) reactions. NR: Not reported. SAR: Sever adverse reaction. ‡ Clinically suspected allergic reactions; § only fatal reaction reported. ¶ Only in six studies as it was not reported/clearly presented in the other three studies. Note: all anaphylaxis and death records were subsets of SARs. **no fatality report mentioned, and we considered it as zero. All anaphylaxis reactions and deaths are subsets (parts) of SARs.

### Pooled incidence of SARs following BPG injection

**Fig 2:**
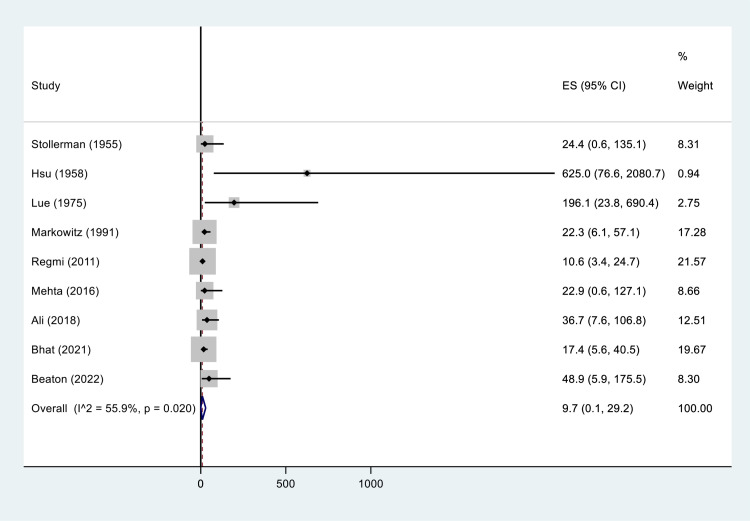
Incidence of SARs following BPG injection, per 10,000 cases.

The overall pooled incidence of SARs following BPG injection for ARF/RHD secondary prophylaxis was 9.7 per 10,000 cases [95% CI: 0.1–29.2, I² = 55.9, P = 0.020] (Fig 2). Subgroup analysis revealed a significant heterogeneity (P = 0.03) in the incidence of SARs across different study designs. Prospective cohort studies reported the higher incidence of SARs at 23.4 per 10,000 cases [95% CI: 0.0–97.6.1], while retrospective cohort studies reported an incidence at 12.9 per 10,000 cases [95% CI: 5.7–22.6] (Table 3).

**Table 3:** Subgroup analysis of incidence of SARs following BPG injection.

Subgroup	Number of studies	Number of RHD patients	Incidence of SAR per 10,000 cases (95% CI)	Overall, DL
**Study design**				
Prospective studies	5	2,770	23.4 (0.0-97.6)	I^2^ = 55.9%, P < 0.001
Retrospective studies	2	7,590	12.9 (5.7-22.6)	
Others^†^	2	1,227	39.5 (9.3-85.8)	
Publication year				
Before 2000	4	2,334	43.7(0.0-182.6)	I2 = 55.9%, P < 0.001
After 2000	5	9,253	15.2(5.1-292.4)	
BPG product				
Lyophilized powder	4	8,817	16.3 (4.8-32.96)	I^2^ = 55.9%, P < 0.001
Not reported	4	2,360	40.9 (0.0-176.22)	

DL: DerSimonian-Laird; ^†^ One was RCT (21), and the other was mixed design (both retrospective and prospective approach) (23).

**Fig 3:**
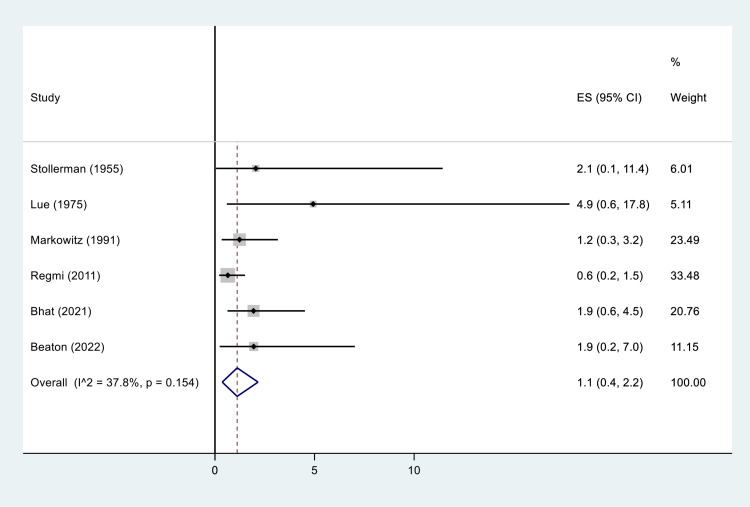
Incidence of SARs per 10,000 BPG injections among ARF and RHD patients.

The overall pooled estimate of SARs incidence per BPG injection, based on six studies (those studies reporting frequency of injections) which comprised 10,301 cases, was 1.1 per 10,000 injections [95% CI: 0.4–2.2, I² = 37.8%, P = 0.154] (Fig 3). Subgroup analysis by study design revealed no significant variation (P = 0.130), with retrospective cohort studies reporting an incidence of 0.9 SARs (95% CI: 0.4–1.6) and non-retrospective studies (three prospective cohorts and one RCT) showing incidence of 1.3 SARs (95% CI: 0.4–2.7) per 10,000 BPG injections (S1 Fig).

**Fig 4:**
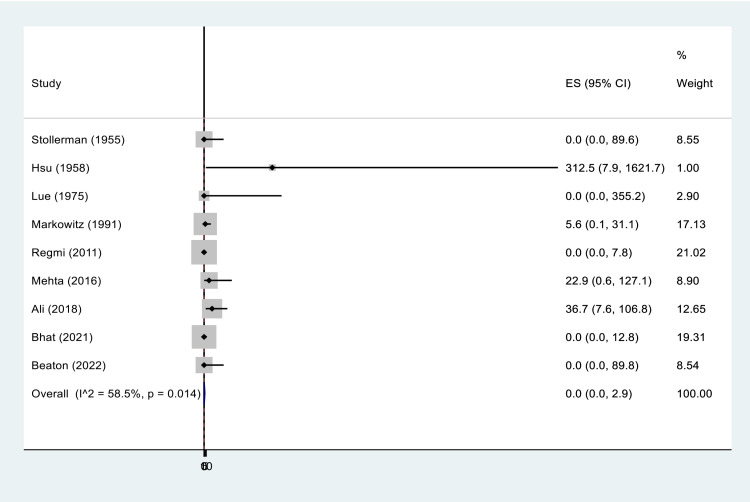
Incidence of fatal reactions following BPG injection per 10,000 cases.

Among the 25 SARs reported, six (24%) were fatal, representing 0.05% of the 11,587 patients receiving BPG for RHD secondary prophylaxis. The overall pooled incidence of fatal reactions following BPG injections were negligible (Fig 4). Notably, all fatal reactions occurred in patients with severe RHD (Table 4).

**Table 4:** Characteristics of patients experiencing fatal reactions following BPG injection.

Case code	Sex	Age (years)	BPG exposure history	Onset of the reaction	Reported type of reaction	Intervention	RHD status
Case 1 [23]	F	15	25^th^	30-60 seconds	Fatal anaphylactic shock	Initial resuscitation and mechanical ventilation for 6 days	Severe mitral valve disease and chronic congestive HF
Case 2 [40]	F	54	20^th^	A Few minutes after	Fatal anaphylactic shock	Epinephrine, Nor-epinephrine, Oxygen via resuscitator	Marked cardiomegaly secondary to aortic regurgitation and stenosis
Case 3 [38]	NR	NR	Received before	NR	Fatal anaphylactic shock	NR	Severe symptomatic RHD
Case 4 [22]	F	6	New	Immediately	Fatal reaction	Proper resuscitation	Severe mitral regurgitation, admitted with severe HF
Case 5 [22]	F	7	Received before	Immediately	Fatal reaction	Proper resuscitation	Severe mitral regurgitation, admitted with severe HF
Case 6 [22]	F	8	Received before	Immediately	Fatal reaction	Proper resuscitation	Severe mitral regurgitation, admitted with severe HF

HF: Heart failure. RHD: Rheumatic heart failure. NR: Not reported.

### Publication bias and sensitivity analysis

Visual inspection of the funnel plot (asymmetrical distribution of studies included in the meta-analysis) indicated the presence of publication bias that may compromise generalizability of the analysis (S2 Fig). Moreover, Egger’s test confirmed the significance of the publication bias (P < 0.05) due to small study effects.

In this meta-analysis, a leave-one-out sensitivity analysis was performed to assess whether the pooled incidence of SARs following BPG administration in patients with RHD/ARF was significantly influenced by any single study. The results indicated that all estimates from the sensitivity analysis remained within the 95% confidence interval of the overall pooled incidence (ranging from 7.0 to 15.2 per 10,000 cases) (Table 5). This finding suggests that no individual study had a disproportionate impact on the observed pooled incidence of SARs following BPG injection in this patient population. Moreover, sensitivity analyses conducted by excluding studies with poor quality scores (Bhat et al. [41] and Regmi et al. [37]) indicated that the incidence of SARs following BPG injection was 20.01 (95% CI: 0.4–57.5) per 10, 000 cases (S3 Fig). Similarly, trimming two studies with small sample sizes (Hsu et al. [40] and Lue et al. [39]), indicated the incidence of SARs following BPG injection was found to be 14.0 (95% CI: 6.7–23.1.7) per 10,000 cases (S4 Fig) and trimming both poor quality and small sample size studies showed an incidence of 25.4 (95% CI: 10.1–46.0) per 10,000 cases (S5 Fig).

**Table 5:** Leave-one-out sensitivity analysis for the incidence of SARs following BPG injection among ARF/RHD patients after removal of each study one at a time.

Study omitted	Incidence of SARs estimate	95% CI
Stollerman (1955)	9.0	0.0-30.3
Hsu (1958)	15.2	4.5-30.2
Lue (1975)	7.3	0.0-22.7
Markowitz (1991)	11.1	0.0-37.1
Regmi (2011)	14.1	0.4-39.9
Mehta (2016)	9.1	0.0-30.6
Ali (2018)	7.6	0.0-28.5
Bhat (2021)	13.6	0.0-42.9
Beaton (2022)	7.0	0.0-26.0

## Discussion

Our systematic review and meta-analysis provide the first systematic effort to quantify the incidence of SARs following BPG injections in patients with ARF/RHD from existing literatures fulfilling the eligibility criteria. The incidence of SARs following BPG injections was rare, between ≥1/10,000 and < 1/1,000 [42]. The lower overall incidence of SARs compared to the individual studies included may be attributable to significant heterogeneity (P < 0.05) [43] among the studies, as well as the influence of larger sample size studies that reported a lower incidence. These larger studies, which demonstrated a lower incidence, were weighted more heavily in the pooled estimate, potentially skewing the overall findings.

Our findings indicate that the incidence of SARs following BPG injections in ARF/RHD patients was higher than the pooled incidence of anaphylaxis reported for the general population receiving BPG, which was 0.2 per 10,000 cases by Galvao et al. [24]. Furthermore, reports by Galvao et al. [24] and Liu et al. [25] each documented only cutaneous adverse reaction in 1,244 and 184 pregnant women, respectively, who were treated with BPG for syphilis. The discrepancy may be attributed to the regular and long-term use of BPG as secondary prophylaxis in ARF/RHD patients, as well as other patient-specific or disease-related factors such as severity of RHD [14,15]. Moreover, in our study non-anaphylactic SARs (n = 6, 24%) were included in the analysis, and there was difference in case definitions.

The incidence of SARs following BPG injection among patients with RHD was comparatively lower than incidence of serious adverse drug reactions, 6.7% [44], and the incidence of SARs, 7.01% [45] in studies encompassing all medical treatments. In contrast, anaphylaxis occurs at an estimated frequency of 1–4 cases per 10,000 courses [46], or 0.015%-0.04% of patients treated with penicillin [47]. These observations underscore the relative infrequency of adverse drug reactions within penicillin-based therapies.

Subgroup analysis by study design revealed that the incidence of SARs in “other” category which included one RCT and one prospective study with a longer follow-up period and some retrospective data and the prospective studies was higher than retrospective studies, with significant heterogeneity among the groups (P < 0.05). The retrospective studies included in the present review had large sample sizes; however, they were based on available medical records (generally in low-income settings), which may have led to the omission of SARs, such as fatal reactions, due to inadequate documentation or under reporting for different reasons [48]. Additionally, the retrospective studies included in the present analysis were conducted after the year 2000. This is further supported by the observed difference in the incidence of SARs between studies conducted before and after 2000 (43.7 Vs 15.2 per 10,000). The decline in SARs may be attributed to the increased availability of higher-quality penicillin products during this period [49]. However, it is important to note that concerns regarding product quality persist [5,6].

In our study, six fatal cases were recorded, representing an overall rate of 0.05%, suggesting that fatalities following BPG injections are rare [42]. When compared to the fatality incidence associated with all drugs in the general population, which ranges from 0.01% to 3.1%, our finding is generally lower [50–52]. However, this rate is notably higher than the 0.0015–0.002% fatality rate observed among patients treated with penicillin for various health problems [47], and no reported fatal outcomes among pregnant women taking BPG for congenital syphilis treatment [24,25]. This suggests there may be an elevated risk of SAR leading to death in individuals with severe RHD and cardiac compromise [14,15].

Notably, our findings reveal a conversion rate of SARs to fatal outcomes at 24%. This stands in stark contrast to existing literature, which reports a fatality rate of only 0.1% for severe adverse drug reactions across a diverse array of medications [50]. Furthermore, approximately 10% of anaphylactic reactions related to penicillin result in fatalities [46]. This discrepancy may be attributable to the predominant oral administration of most penicillins, as opposed to the injectable BPG, which is associated with a comparatively lower risk of severe reactions in addition to disease related factors. Additionally, because the treatment is injectable, there is a risk of injection site errors, as demonstrated by an incident of sciatic nerve injury documented in our study [20]. This highlights the importance of addressing gaps in intramuscular injection skills through targeted training.

Remarkably, most fatal reactions were observed in females and children, which might be attributed to the relatively high burden of RHD among children and adolescents and females compared to adults and males [2]. Another possible explanation is the growing evidence from recent studies suggesting an increased risk of penicillin allergy associated with female sex [53,54]. Additionally, with the exception of one study [40] all included studies focused on paediatric populations or at least included children alongside adults, which may further influence these findings.

Moreover, all six reported fatalities occurred in patients with severe RHD, which can be categorized under high risk according to the American Heart Association Advisory Panel risk stratification for death due to vasovagal compromise [14]. The emerging notion [14,15] is that this may be due to the underlying pathophysiological condition, which could impair the body’s ability to handle vasovagal reactions, pain shock, or anaphylaxis related to BPG injection, possibly due to cardiac compromise or other disease/patient-specific factors. In the majority of fatal cases, resuscitation efforts were initiated despite unsuccessful recovery, suggesting that the adverse reaction was immediate and/or the administration of adrenaline may have contributed to a worsened outcome by increasing cardiac workload and exacerbating the underlying conditions in patients with advanced heart failure [55]. However, the mechanisms underlying mortality in this population warrant further investigation along with a well-designed adverse reaction reporting system for BPG.

Given that vasovagal reactions associated with the painful intramuscular injection seems to be a key contributing factor to fatal reactions in patients with severe RHD (i.e., cardiac compromise) [14,15], future research should focus on optimizing BPG formulations, delivery strategies and mitigation approaches. Given the well-established clinical benefits of BPG [21], enhancing its safety profile could significantly mitigate the risk of SARs following intramuscular administration. Additionally, tailored strategies for RHD patients may be essential to minimize both the risk of adverse reactions and the reluctance of healthcare professionals to administer BPG, thereby ensuring broader and more effective delivery in this high-risk population.

Our systematic review has several limitations. First, the exclusion of non-English studies may have restricted the comprehensiveness of our findings, as relevant insights from non-English literature could not be incorporated due to resource and language constraints. Additionally, some included studies were not primarily designed to assess SARs to BPG, resulting in reduced statistical power to detect rare events, thereby limiting the ability to determine the true incidence of SARs [56]. Potential underreporting of adverse reactions and incomplete data further constrained the reliability and generalizability of our findings. Another limitation was the inability to compare the incidence of SARs between the two known forms of BPG; the powdered injection and the suspension in pre-filled syringes (normally used in some developed countries and settings with reliable cold-chain logistics). Furthermore, subgroup analysis of SAR incidence among patients with severe versus moderate/mild RHD was not possible due to insufficient clinical information in the source studies. Finally, although meta-analysis statistically combines data from two or more studies [32], the relatively small number of studies included in this review may affect the interpretation and reliability of results, particularly in subgroup analyses.

## Conclusion

In conclusion, our systematic review and meta-analysis found that SARs to BPG injections in patients with ARF and RHD are rare. However, due to the limited statistical power of included studies, larger, well-powered studies are needed to confirm these findings and refine incidence estimates. These findings support the continued use of BPG for secondary prophylaxis in high-risk populations, emphasizing the importance of balancing the very low risk of SARs with the significant benefits of preventing disease progression. Nonetheless, the risk of SARs following BPG injection should not be overlooked, given their severity and the substantial proportion that can result in fatal outcomes. Statistically powered, high-quality longitudinal research and comprehensive adverse reaction reporting at large is necessary to strengthen the evidence, as healthcare providers and patients frequently express safety concerns that impact the delivery of BPG.

## Supporting information

S1 Checklist PRISMA Checklist. (DOCX)

S1 Data List of all literatures with reason for exclusion. (XLSX)

S1 File Search strategy. (DOCX)

S2 File Quality assessment of cohort studies. (DOCX)

S3 File Cochrane risk of bias assessment. (DOCX)

S1 Fig Subgroup analysis by study design of incidence of SARs per 10,000 BPG injections. (DOCX)

S2 Fig Funnel plot assessing distribution of included studies for SARs incidence. (DOCX)

S3 Fig Incidence of SARs per 10,000 cases after trimming poor quality studies. (DOCX)

S4 Fig Incidence of SARs per 10,000 cases after trimming low sample size studies. (DOCX)

S5 Fig Incidence of SARs after trimming both poor quality and small sample size studies. (DOCX)

S4 File Raw meta-analysis datasets. (DOCX)
