# Supplementary material for: Severe adverse reactions to benzathine penicillin G in rheumatic heart disease: A systematic review and meta-analysis
Source: PLoS One. 2025 May 7;20(5):e0322873. doi: 10.1371/journal.pone.0322873 (PMC12057857; doi:10.1371/journal.pone.0322873)
Supplement: S1 File — (DOCX) [file pone.0322873.s003.docx]

**Supplementary Table 2: Search strategy**

PubMed Searched on 6 July 2024

| Search | Theme | Keywords | Results |
| --- | --- | --- | --- |
| #1 | Adverse reactions | "reaction*"[Title/Abstract] OR "event*"[Title/Abstract] OR "adverse*"[Title/Abstract] OR "anaphylaxis"[Title/Abstract] OR "serious"[Title/Abstract] OR "severe"[Title/Abstract] OR "Fatal"[Title/Abstract] OR "Sudden"[Title/Abstract] OR "death*"[Title/Abstract] OR "shock*"[Title/Abstract] OR "effect*"[Title/Abstract] OR "Anaphylactic"[Title/Abstract] OR "allerg*"[Title/Abstract] OR "Safety"[Title/Abstract] OR "outcome*"[Title/Abstract] | 14,059,963 |
| # 2 | Benzathine penicillin G | ("penicillin g benzathine"[MeSH Terms] OR "benzathine penicillin g"[Title/Abstract] OR "benzylpenicillin benzathine"[Title/Abstract] OR "penicillin g benzathine"[Title/Abstract] OR (("benzylpenicillins"[All Fields] OR "penicillin g"[MeSH Terms] OR "penicillin g"[All Fields] OR "benzylpenicillin"[All Fields] OR "penicilline"[All Fields] OR "penicillines"[All Fields] OR "penicillins"[MeSH Terms] OR "penicillins"[All Fields] OR "penicillin"[All Fields]) AND "benzathine benzyl"[Title/Abstract]) OR "benzathine benzylpenicillin"[Title/Abstract] OR "benzathine penicillin"[Title/Abstract] OR "bicillin l-a"[Title/Abstract] OR "permapen"[Title/Abstract] OR "penadur l-a"[Title/Abstract] OR "extencillin"[Title/Abstract] OR ("g"[All Fields] AND "benzatinica"[Title/Abstract]) OR "penicillin g"[Title/Abstract] OR "benzathine"[Title/Abstract] OR "penicillin"[Title/Abstract] OR "benzylpenicillin g"[Title/Abstract] OR "benzylpenicillin"[Title/Abstract] OR "benzathine benzylpenicillin"[Title/Abstract] OR (("benzylpenicillins"[All Fields] OR "penicillin g"[MeSH Terms] OR "penicillin g"[All Fields] OR "benzylpenicillin"[All Fields] OR "penicilline"[All Fields] OR "penicillines"[All Fields] OR "penicillins"[MeSH Terms] OR "penicillins"[All Fields] OR "penicillin"[All Fields]) AND "g benzathine"[Title/Abstract]) OR "bicillin"[Title/Abstract] OR "BPG"[Title/Abstract]) | 63,152 |
| # 3 | Rheumatic fever heart disease | "Rheumatic fever"[MeSH Terms] OR "rheumatic fever"[Title/Abstract] OR "Acute Rheumatic Fever"[Title/Abstract] OR "rheumatic fever acute"[Title/Abstract] OR "rheumatic fever"[Title/Abstract] OR "Rheumatic Heart Disease"[MeSH Terms] OR "Rheumatic Heart Disease"[Title/Abstract] OR "Rheumatic Heart Diseases"[Title/Abstract] OR "Rheumatic Carditis"[Title/Abstract] OR "Chronic Rheumatic Heart Disease"[Title/Abstract] OR "Rheumatic Valve Disease"[Title/Abstract] | 26,163 |
| #4 | | #1 AND #2 AND #3 | 438 |
| Limited | | Human | 377 |
|  |  | Human and English | 308 |

Scopus Searched on 6 July 2024

| Search | Theme | Keywords | Results |
| --- | --- | --- | --- |
| #1 | Adverse reactions | TITLE-ABS("reaction*" OR "event*" OR "adverse*" OR "anaphylaxis" OR "serious" OR "severe" OR "fatal" OR "sudden" OR "death*" OR "shock*" OR "effect*" OR "anaphylactic" OR "allerg*" OR "safety" OR "outcome*") | 31,698,158 |
| # 2 | benzathine penicillin G | (TITLE-ABS("penicillin g benzathine" OR "benzathine penicillin g" OR "benzylpenicillin benzathine" OR "penicillin g benzathine" OR "benzathine benzylpenicillin" OR "benzathine penicillin" OR "bicillin l-a" OR "permapen" OR "penadur l-a" OR "extencillin" OR "penicillin g" OR "benzathine" OR "penicillin" OR "benzylpenicillin g" OR "benzylpenicillin" OR "bicillin" OR "BPG") AND ALL("benzathine benzyl" OR "benzatinica" OR "g benzathine")) | 1,144 |
| # 3 | Rheumatic fever heart disease | (TITLE-ABS("rheumatic fever" OR "acute rheumatic fever" OR "rheumatic fever acute" OR "rheumatic heart disease" OR "rheumatic heart diseases" OR "rheumatic carditis" OR "chronic rheumatic heart disease" OR "rheumatic valve disease")) | 15,486 |
| #4 | | #1 AND #2 AND #3 | 91 |
| Limited | | English | 82 |

Web of Science Searched on 6 July 2024

| Search | Theme | Keywords | Results |
| --- | --- | --- | --- |
| #1 | Adverse reactions | TS=("reaction*" OR "event*" OR "adverse*" OR "anaphylaxis" OR "serious" OR "severe" OR "fatal" OR "sudden" OR "death*" OR "shock*" OR "effect*" OR "anaphylactic" OR "allerg*" OR "safety" OR "outcome*") | 26,063,437 |
| # 2 | benzathine penicillin G | TS=("penicillin g benzathine" OR "benzathine penicillin g" OR "benzylpenicillin benzathine" OR "penicillin g benzathine" OR "benzathine benzylpenicillin" OR "benzathine penicillin" OR "bicillin l-a" OR "permapen" OR "penadur l-a" OR "extencillin" OR "penicillin g" OR "benzathine" OR "penicillin" OR "benzylpenicillin g" OR "benzylpenicillin" OR "bicillin" OR "BPG") AND TS=("benzathine benzyl" OR "benzatinica" OR "g benzathine") | 123 |
| # 3 | Rheumatic fever heart disease | TS=("rheumatic fever" OR "acute rheumatic fever" OR "rheumatic fever acute" OR "rheumatic heart disease" OR "rheumatic heart diseases" OR "rheumatic carditis" OR "chronic rheumatic heart disease" OR "rheumatic valve disease") | 9,327 |
| #4 | | #1 AND #2 AND #3 | 14 |
| Limited | | English | 14 |
