# Supplementary material for: Severe adverse reactions to benzathine penicillin G in rheumatic heart disease: A systematic review and meta-analysis
Source: PLoS One. 2025 May 7;20(5):e0322873. doi: 10.1371/journal.pone.0322873 (PMC12057857; doi:10.1371/journal.pone.0322873)
Supplement: S2 File — (DOCX) [file pone.0322873.s004.docx]

**Supplementary Table 3: Quality assessment of cohort studies included in this study based on Newcastle-Ottawa scale**

| **Study Name** | **Selection** | | | | **Comparability** | **Outcome** | | | **Total (9⋆)** |  |
| --- | --- | --- | --- | --- | --- | --- | --- | --- | --- | --- |
|  | Representativeness of exposed cohort (⋆) | Selection of non-exposed cohort (⋆) | Ascertainment of exposure (⋆) | Demonstrate that outcome of interest was not present at start of study (⋆) | Comparability (⋆⋆) | Assessment of outcome (⋆) | Follow-up long enough for outcomes to occur (⋆) | Adequacy of follow-up of cohorts (⋆) |  |  |
| Markowitz, 1991 | * |  | * | * |  | * | * | * | 6 |  |
| Bhat et al., 2021 | * |  | * |  |  | * |  |  | 3 |  |
| Stollerman et al., 1955 | * |  | * | * |  | * | * | * | 6 |  |
| Hsu et al., 1958 | * |  | * | * |  | * | * | * | 6 |  |
| Mehta et al 2016 |  |  | * | * |  | * | * | * | 5 |  |
| Lue et al., 1975 | * |  | * | * |  | * | * | * | 6 |  |
| Ali et al., 2018 | * |  | * |  |  | * | * | * | 5 |  |
| Regmi et al., 2011 | * |  | * |  |  | * |  |  | 3 |  |

*Source* *Peterson J, Welch V, Losos M, Tugwell P. 2011. The Newcastle-Ottawa scale (NOS) for assessing the quality of nonrandomised studies in meta-analyses.*

**Supplementary Table 4: Risk of bias assessment:** For Beaton et al 2022 based on Cochrane risk of bias assessment


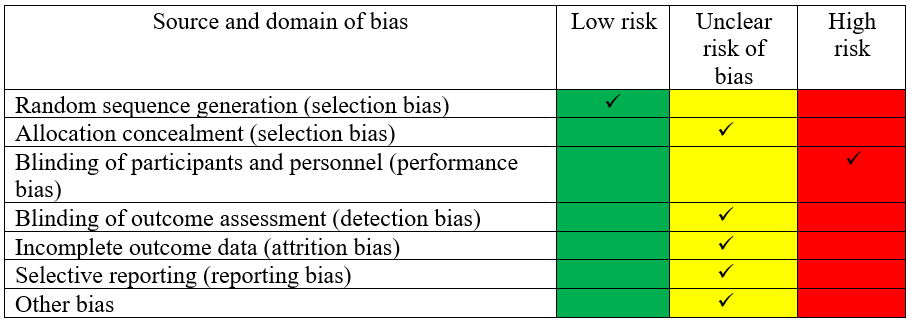


*Source: Higgins JP, Altman DG, Gøtzsche PC, et al. The Cochrane Collaboration’s tool for assessing risk of bias in randomised trials. BMJ. 2011;343.*

1 = non-retrospective, 0 = retrospective studies

**Supplementary Fig. 1: Subgroup analysis by study design of incidence of SARs per 10,000 BPG injections**


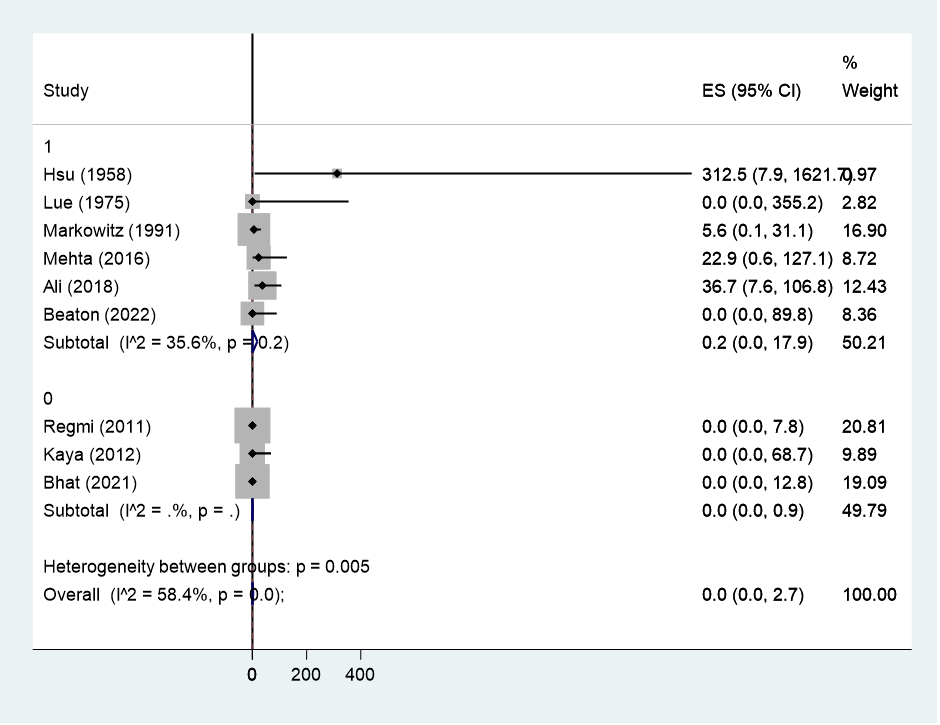


1 = Moderate quality cohorts including the RCT, 0 = Poor quality (retrospective cohorts)

**Supplementary Fig. 2: Subgroup analysis of incidence of fatality by study quality**

ES: Effect size

**Supplementary Fig. 3: Funnel plot assessing distribution of included studies for SARs incidence**

**Supplementary Fig. 4: Incidence of SARs per 10,000 cases after trimming poor quality studies**

**Supplementary Fig. 5: Incidence of SARs per 10,000 cases after trimming low sample size studies**

**Supplementary Fig. 6: Incidence of SARs after trimming both poor quality and small sample size studies**
