# Supplementary material for: Severe adverse reactions to benzathine penicillin G in rheumatic heart disease: A systematic review and meta-analysis
Source: PLoS One. 2025 May 7;20(5):e0322873. doi: 10.1371/journal.pone.0322873 (PMC12057857; doi:10.1371/journal.pone.0322873)
Supplement: S3 File — (DOCX) [file pone.0322873.s005.docx]

**Supplementary Table 4: Risk of bias assessment:** For Beaton et al 2022 based on Cochrane risk of bias assessment


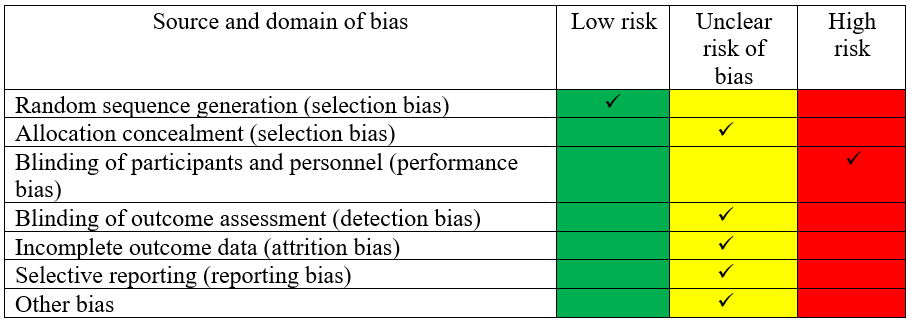


*Source: Higgins JP, Altman DG, Gøtzsche PC, et al. The Cochrane Collaboration’s tool for assessing risk of bias in randomised trials. BMJ. 2011;343.*
