# Supplementary material for: Severe adverse reactions to benzathine penicillin G in rheumatic heart disease: A systematic review and meta-analysis
Source: PLoS One. 2025 May 7;20(5):e0322873. doi: 10.1371/journal.pone.0322873 (PMC12057857; doi:10.1371/journal.pone.0322873)
Supplement: S2 Fig — (DOCX) [file pone.0322873.s007.docx]

ES: Effect size

**S2 Fig: Funnel plot assessing distribution of included studies for SARs incidence**
