# Supplementary material for: Severe adverse reactions to benzathine penicillin G in rheumatic heart disease: A systematic review and meta-analysis
Source: PLoS One. 2025 May 7;20(5):e0322873. doi: 10.1371/journal.pone.0322873 (PMC12057857; doi:10.1371/journal.pone.0322873)
Supplement: S5 Fig — (DOCX) [file pone.0322873.s010.docx]

**S5 Fig: Incidence of SARs after trimming both poor quality and small sample size studies**
