# Supplementary material for: Severe adverse reactions to benzathine penicillin G in rheumatic heart disease: A systematic review and meta-analysis
Source: PLoS One. 2025 May 7;20(5):e0322873. doi: 10.1371/journal.pone.0322873 (PMC12057857; doi:10.1371/journal.pone.0322873)
Supplement: S4 File — (DOCX) [file pone.0322873.s011.docx]

**Raw Meta-analysis datasets**

| **S/no** | **First Author** | **Year** | **Enrolment/review/Data Collection period** | **Country** | **Setting** | **Population** | **Regimen** | **Design** | **Sample size (by RHD patient taking BPG)** | **Follow up duration** | **Sample size (by BPG injections)** | **Age** | **Sex** | **Brand of BPG** |
| --- | --- | --- | --- | --- | --- | --- | --- | --- | --- | --- | --- | --- | --- | --- |
| 1 | Markowitz | 1991 | 1988-1990 | 11 countries | Not reported | Adults and children with rheumatic fever and on BPG secondary prophylaxis | 1.2 MIU, IM every 4 week | Prospective Cohort | 1790 | 6-24 months | 32,430 | Their ages ranged from 5-28 years; 33% were 12 years or younger | Not reported | Not reported |
| 2 | Bhat | 2021 | Medical records from 2015-2020 | India | cardiac care | All outpatient RHD cases | 0.6MIU for under 27kg and 1.2MIU for over 30kg every 3 week | Retrospective cohort | 2878 | N/A as it is retrospective study | 25,819 | The mean age = 31.54 ± 10.76 years. | 41.5% male | Lyophilized powder |
| 3 | Beaton | 2022 | 2018 -2020 | Uganda | Cardiac clinic based | Children and adolescents, 1ged 5-17 years | 1.2MIU for >30kg and 0.6 for <30kg/month | Other | 409 | 24 months/2 years | 10,284 | Mean=12.6±2.8 | 43.0 male | Lyophilized powder |
| 4 | Hsu | 1958 | 1955-1957 | USA | Hospital based | Adults with RHD | 1.2MIU/month | Prospective Cohort | 32 | 1 month-27 months | Not reported | 20-54 years | Not reported | Not reported |
| 5 | Mehta | 2016 | 2012-2015 | India | Cardiac centres | Children and adolescents, 1ged 5-17 years | 0.6MIU for under 27kg and 1.2MIU for over 30kg every 3-4 week | Prospective Cohort | 436 | 11 Months (median) (6-18 months inter-quartile range 6-18) | Not reported | 12.2±3.2 | 66.1% male | Not reported |
| 6 | Lue | 1975 | 1967-1971 | Taiwan | Institution based | Children <18 years | 1.2MIU/month | Prospective Cohort | 102 | 6 months - 6 years duration of prophylaxis (average 4.4 years) | 4,056 | <18 years old | 56% male | Not reported |
| 7 | Ali | 2018 | 2005-2018 | Sudan | 2 referral hospitals | Children <18 years | 0.6MIU for <7 years and 1.2MIU for >7 years/3week | Other | 818 | 1-10 years | Not reported | 3-18 years | 51% male | Lyophilized powder |
| 8 | Regmi | 2011 | Medical records from2007 -Feb 2010 | Nepal | 32 hospitals | children and adults | 0.6MIU for <30kg and 1.2MIU for >30KG every 3 week | Retrospective cohort | 4712 | N/A as it is retrospective study | 77,300 | <18 years old (36.7%) | 46.1% male | Lyophilized powder |
| 9 | Stollerman | 1955 | 1952-1954 | USA | Hospital based | Children | 1.2MIU per month | Prospective Cohort | 410 | 1-2 years | 4,871 | 6-16 years | Not reported | Bicillin |

**Allergic reactions, SARs, anaphylaxis and fatal reactions**

| **S/no** | **First Author** | **Year** | **Allergic reactions (Clinically reported or suspected cases)** | **SARs** | **Anaphylaxis** | **Death** |  |
| --- | --- | --- | --- | --- | --- | --- | --- |
| 1 | Markowitz | 1991 | 57 | 4 | 4 | 1 |  |
| 2 | Bhat | 2021 | 7 | 5 | 5 | 0 |  |
| 3 | Beaton | 2022 | 8 | 2 | 1 | 0 | No fatal reaction documented, and we considered it as 0 |
| 4 | Hsu | 1958 | 6 | 2 | 1 | 1 |  |
| 5 | Mehta | 2016 | 1 | 1 | 1 | 1 |  |
| 6 | Lue | 1975 | 7 | 2 | 2 | 0 | No fatal reaction documented, and we considered it as 0 |
| 7 | Ali | 2018 | Not report | 3 | 0 | 3 |  |
| 8 | Regmi | 2011 | 66 | 5 | 5 | 0 | No fatalities specified and we considered it as 0 |
| 9 | Stollerman | 1955 | 5 | 1 | 0 | 0 | No fatalities specified and we considered it as 0 |
